# Terahertz 3D bulk metamaterials with randomly dispersed split-ring resonators

**DOI:** 10.1515/nanoph-2021-0703

**Published:** 2022-02-28

**Authors:** Taiyu Okatani, Yuto Sunada, Kazuhiro Hane, Yoshiaki Kanamori

**Affiliations:** Department of Robotics , Graduate School of Engineering, Tohoku University, 6-6-01 Aoba, Aramaki, Aoba-ku, Sendai, Miyagi, 980-8579, Japan; Department of Finemechanics, Graduate School of Engineering, Tohoku University, 6-6-01 Aoba, Aramaki, Aoba-ku, Sendai, Miyagi, 980-8579, Japan

**Keywords:** 3D metamaterial, bulk metamaterial, metamateria, split-ring resonator, terahertz

## Abstract

While optical systems using terahertz wave are expected to achieve beneficial applications, at present, the materials of the optical elements that compose them must be selected from limited choices. In this study, we propose a three-dimensional bulk metamaterial in which metal microstructures are dispersed in the bulk resin randomly. A bulk metamaterial was designed and fabricated, in which split-ring resonators known as typical metamaterials were dispersed in cyclo-olefin polymer. In the fabrication method, a resin sheet containing split-ring resonators was first prepared and then diced into resin grains containing a single split-ring resonator. Finally, they were filled in a mold and solidified with a resin solution to obtain the target bulk metamaterial. The optical properties of the fabricated bulk metamaterial were measured by terahertz time-domain spectroscopy. The measurement results confirmed that the refractive index deviated from the original refractive index of the cyclo-olefin polymer due to the resonance of split-ring resonators, suggesting that the proposed bulk metamaterials could be used as a new optical material in the terahertz band.

## 1 Introduction

Terahertz wave is an electromagnetic wave at a frequency of approximately 0.1–10 THz. Although terahertz wave has been regarded as an undeveloped electromagnetic wave until recent years, known as ‘terahertz gap’, technologies for generating and detecting terahertz wave have been rapidly developed, and research and development for their applications are being actively carried out [1]. Terahertz wave penetrates various substances other than metals relatively easily. In addition, since the terahertz band contains the vibration-rotation spectra of several molecules, terahertz wave can be used to analyze the properties of substances. Furthermore, since the photon energy of terahertz wave is lower than that of X-rays, there is little adverse effect on the living body. Due to these characteristics, it is expected that chemical analysis and nondestructive inspection using terahertz spectroscopy will be realized [2], [3], [4], [5], [6], [7], [8], [9], [10], [11]. For example, it has been reported that three types of illicit drugs hidden in mail envelopes can be identified from the difference in the absorption spectrum in the terahertz band called the fingerprint spectrum [12]. The fingerprint spectrum is not seen in the radio band with a wavelength longer than terahertz wave. In the light region with a wavelength shorter than the terahertz wave, the transmission is lower even if the fingerprint spectrum appears. Therefore, the effective use of terahertz wave may expand applications that were previously impossible with electromagnetic waves.

One of the major challenges in effectively utilizing terahertz wave is the lack of natural materials that can be used as optical elements such as lenses, prisms, mirrors, and filters, which are commonly used in the visible light region. There are many types of optical glasses in the visible light region, and various material properties such as refractive index, Abbe number, relative partial dispersion, and coefficient of thermal expansion can be selected from a wide range of choices according to the application [13]. Furthermore, by combining them with optical thin films made of dielectrics or metals, various filters and mirrors can be realized. On the other hand, there are only a few materials currently used as optical elements in the terahertz band [14]. Unfortunately, some optical materials often used in the visible light region are unsuitable as materials for optical elements in the terahertz band. For example, silicate glass, which shows high transparency in the visible light region, shows strong absorption in the terahertz band [15]. Thus, the search for optical materials in the terahertz band is underway. One typical example suitable as a terahertz optical material is float-zone silicon (FZ-Si), which is high-purity crystalline silicon with high electric resistivity and high transparency in the terahertz band [16]. Lenses and prisms made by polishing FZ-Si can be used for constructing precise terahertz optics because of their low dispersion over a wide frequency band. Other crystalline materials used as terahertz optical materials include crystalline quartz, sapphire, and synthetic diamond. These crystalline materials possess relatively high refractive indices although they are difficult to process into arbitrary shapes. Several polymers can be used as alternative materials such as cyclo-olefin polymer (COP), polymethylpentene, high-density polyethylene, polypropylene, and polytetrafluoroethylene [17]. These polymers show high transmittance in the terahertz band and are easy-to-process.

As mentioned above, some terahertz optical materials are known and commercially available. However, the range of choices is narrow compared to optical materials used in the visible light region. In particular, the range of refractive indices that can be selected is limited. It is difficult to control the refractive index of crystalline materials such as FZ-Si by changing their composition like for optical glasses. In the case of polymers, the composition and density can be changed relatively easily although it is difficult to obtain a high refractive index. A wide range of choices in refractive index and its dispersion are essential in designing and manufacturing high-performance optical elements. For example, imaging lenses with low aberrations and spectroscopic prisms with high aberrations will be required for applications using terahertz wave such as chemical analysis and nondestructive inspection. If there is no material in nature that meets the needs, it is necessary to artificially create a material with optimum optical properties.

One possibility that may solve this problem is metamaterials. Metamaterials are artificial optical materials that use the interaction between light and a structure smaller than its wavelength to exhibit optical properties not found in natural materials. Depending on the design of the microstructure, the effective permittivity of the medium containing the structure, as well as the magnetic permeability, which should normally be 1 in the light region, can be controlled to the desired value [18]. Taking advantage of this feature, various metamaterial-based applications such as sensors [19], [20], [21], [22], [23], [24], [25], modulators for phase, amplitude, frequency, or polarization [26], [27], [28], [29], [30], [31], absorbers [32, 33], wavelength-selective filters [34, 35], quarter-wave plate [36], and light emitting elements [37] have been shown. Moreover, tunable metamaterials in which the microstructure is movable using micro-electro-mechanical systems [38], [39], [40], [41] and applications to quantum photonics [42] are also attracting attention. A typical structure known as a metamaterial is a split-ring resonator (SRR) [43]. The SRR has a shape in which a part of the metal ring is cut off, and the effective magnetic permeability can be controlled by the magnetic field generated by the orbital current flowing through the ring. Well-designed SRRs are known to exhibit a negative permeability and a negative refractive index when combined with metamaterials that exhibit a negative permittivity [44]. In the terahertz band, in addition to a negative refractive index, a zero refractive index and ultrahigh refractive index have also been reported for metamaterials made from cut-wire pairs [45], [46], [47]. Furthermore, while natural materials with high refractive indices show high Fresnel reflections, metamaterials can achieve low reflection and high refractive index simultaneously by controlling their impedances [45], [46], [47] or refractive index distribution [48]. The realization of exotic optical properties such as a negative refractive index enables extremely innovative optical elements represented by perfect lenses or superlenses [49, 50], which are also expected to be useful in terahertz optics.

Although metamaterials may provide design freedom for refractive index and solve the shortage of optical materials in the terahertz band, many of them reported thus far are not bulk materials like natural materials that make up ordinary lenses and prisms but two-dimensional (2D) materials like thin films, in which the microstructures are periodically arranged on the surface of the substrate. In the case of 2D metamaterials, often referred to as metasurfaces [51], the desired responses are designed by placing microstructures of appropriate shape and dimensions at appropriate locations on the substrate. Metalenses are a well-known example of optical elements that use metasurfaces [52]. While metasurfaces can ultimately provide thin optical elements compared to bulk materials, the interaction distance with the electromagnetic wave may be too short to obtain sufficient performance because the interaction occurs within only one layer of metasurfaces. For this reason, several attempts have been made to create three-dimensional (3D) bulk metamaterials in which microstructures are superimposed in the thickness direction [53]. In some studies, multiple layers of metasurfaces created using photolithography have been used to obtain 3D metamaterials [54, 55]. For example, Han et al. have succeeded in stacking polyethylene naphthalate films on which SRRs are collectively formed by photolithography [55]. Using photolithography, it is possible to design and fabricate complex microstructures that cannot be created by self-assembly [56]. Furthermore, fabrication methods using photolithography are more productive than other methods such as electron-beam lithography [57] and direct laser writing [58], and it is relatively easy to fabricate multiple layers. However, it is necessary to prepare each layer and stack the layers with alignment, which is not easy in terms of productivity. In addition, 3D structures made by stacking metasurfaces are generally anisotropic and exhibit dependency on the incident direction and polarization, which can be a constraint in designing optical elements. Although anisotropy may be eliminated by staking metasurfaces with out-of-plane microstructures [59], [60], [61], it is generally difficult to create out-of-plane microstructures by photolithography, and completely isotropic metamaterials have not been realized yet.

**Figure 1:**
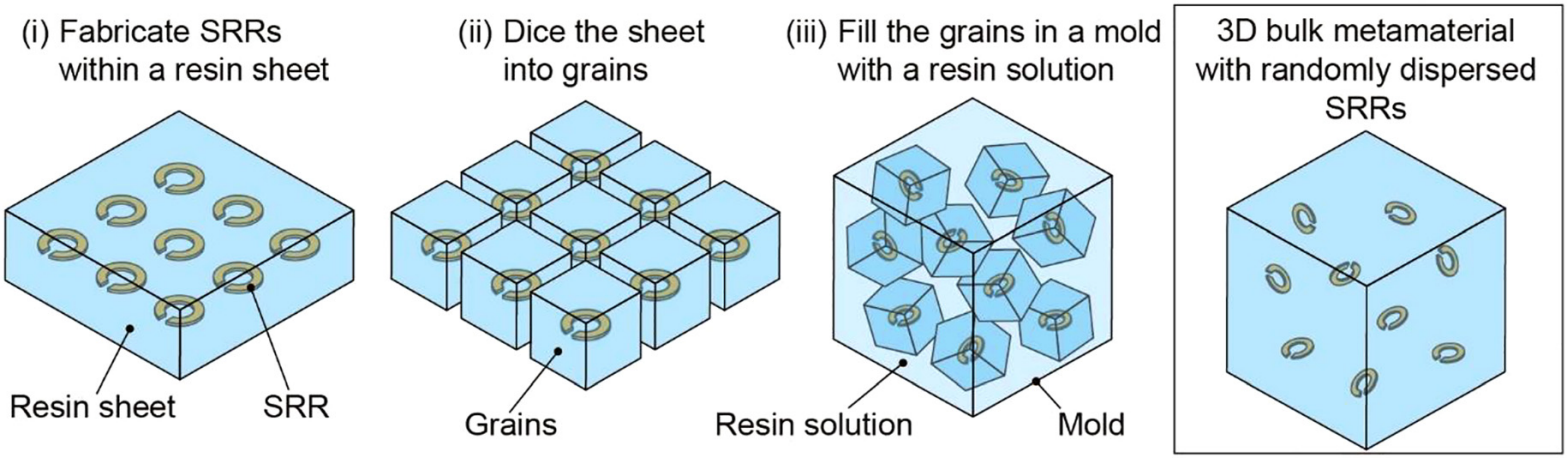
Conceptual sketch of the proposed 3D bulk metamaterial with randomly dispersed SRRs and its fabrication processes.

In this study, aiming at optical materials in the terahertz band, we propose isotropic 3D bulk metamaterials (Figure 1). By dispersing the microstructures that make up the metamaterial in random directions, isotropic optical properties are achieved. The proposed fabrication method is completely different from the conventional layer stacking methods and is easier. SRRs designed to respond in the terahertz band are selected as the microstructures contained in the bulk metamaterial. First, a resin sheet containing SRRs is fabricated. Second, the sheet is diced into grains, each of which contains an SRR. Third, the grains are filled in a mold with a solution diluting resin. Finally, by solidification, a 3D bulk metamaterial with randomly dispersed SRRs is obtained. In the proposed fabrication method, the microstructures are dispersed while being covered with resin; thus, the optical properties of the metamaterials are never lost by the agglutination of microstructures. Furthermore, by changing the size of the resin grains containing the microstructures during the dicing process, the density of the microstructures in the bulk metamaterial can be controlled. After fabricating the prototype of the bulk metamaterial, the optical properties are measured to verify that the anisotropy seen in the metasurface is eliminated in the 3D bulk metamaterial.

## 2 Design and fabrication

### 2.1 Design and simulation

**Figure 2:**
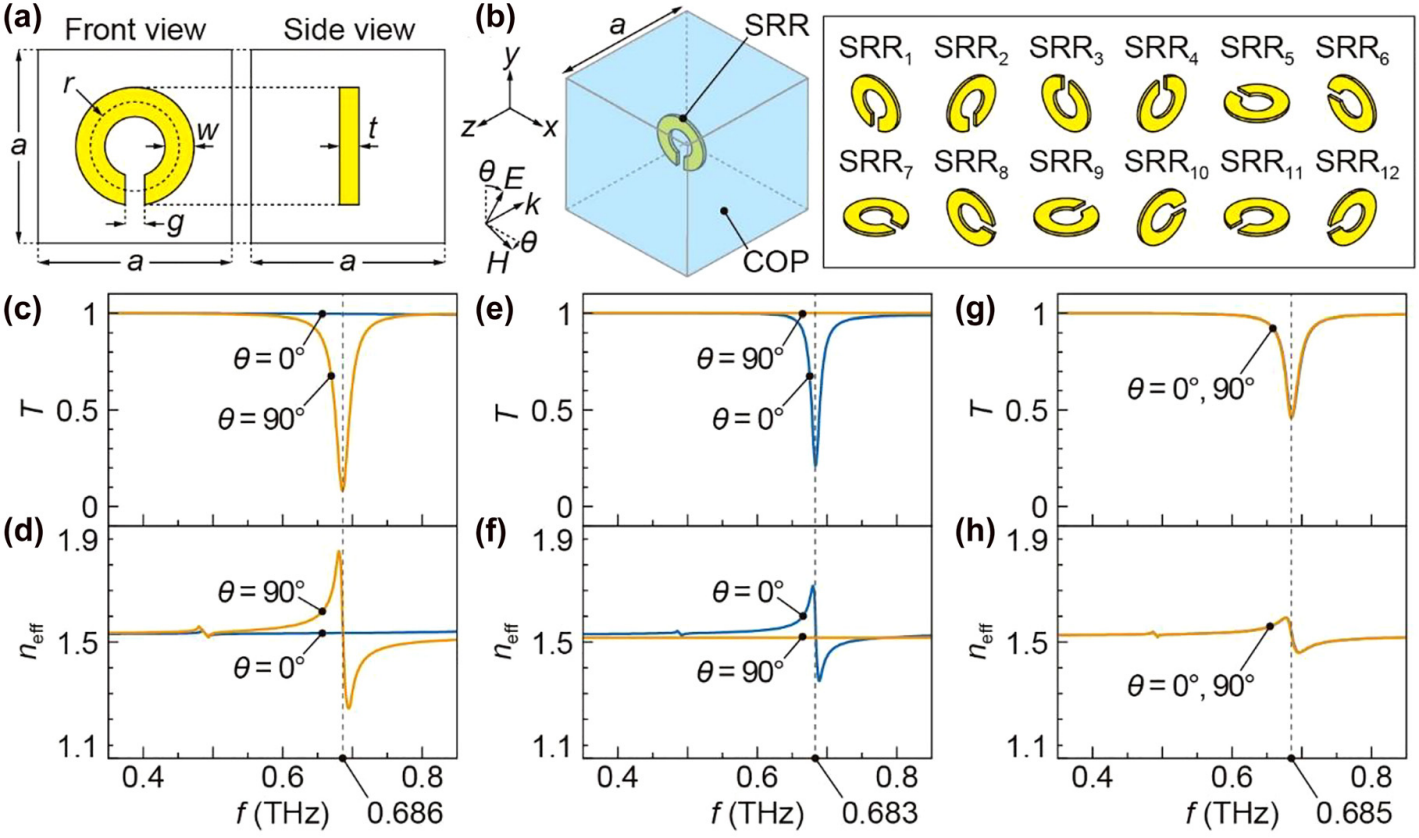
(a) Design of a resin grain containing a single SRR. (b) The unit cell for a calculation model containing one of the 12 types of differently-directed SRRs from SRR_1_ to SRR_12_. (c) and (d) Simulation results of the transmittance and effective refractive index for a calculation model containing SRR_1_. (e) and (f) Simulation results for a calculation model containing SRR_2_. (g) and (h) The transmittance and effective refractive index obtained from the average *S* parameters of 12 types of SRRs. The results with *x*-axis polarized wave (*θ* = 90°) and *y*-axis polarized wave (*θ* = 0°) almost overlap.

To design a bulk metamaterial containing SRRs, the optical properties were calculated using electromagnetic field simulation software (CST Studio Suite, Dassault Systèmes S.E.). Figure 2(a) shows a resin grain containing a single SRR. The grain was a cube with a side length of *a*, and an SRR was located in the center of the grain. The radius, line width, gap width, and thickness of an SRR were defined as *r*, *w*, *g*, and *t*, respectively. Gold was selected as the material for the SRR. The material of the resin sheet was assumed to be COP, and its dielectric constant was set to 2.3 to correspond to the square of the refractive index of COP, 1.525 [17]. The grains can be obtained by dicing a resin sheet containing SRRs. Note that the distance between the centers of adjacent SRRs in the state of the resin sheet does not match the size of the grain due to the kerf width in the dicing process; in other words, the resin sheet should be designed with the kerf width in mind.

Since a single SRR shows a different response when its direction to the incident electromagnetic field changes, the resin sheet containing only SRRs in the same direction has polarization dependence. In the target 3D bulk metamaterial, by filling the grains in a mold with a resin solution, SRRs in various directions are randomly dispersed in the bulk resin; therefore, it is expected that the bulk metamaterial will show a response that does not depend on the incident polarization angle. To predict the optical properties of the bulk metamaterial, simulations were performed on a calculation model that included an SRR in various directions. Figure 2(b) shows the unit cell of the calculation model. The unit cell contained one of the 12 types of differently directed SRRs from SRR_1_ to SRR_12_ in a cube made of COP with a side length of *a*. The incident wave was heading in the −*z*-axis, and the incident polarization angle (*θ*) was defined to be zero degrees when the electric field was parallel to the *y*-axis. The 12 types of SRRs cover all the attitudes obtained when a combination of 90-degree rotations around the *x*-axis, *y*-axis, and *z*-axis is applied to a grain containing an SRR shown in Figure 2(a). Therefore, assuming that the 12 types of grains are randomly arranged in the bulk metamaterial and that the *S* parameters for reflection and transmission of the bulk metamaterial (*S*_11,bulk_, *S*_21,bulk_) are equal to the average of those of all the attitudes, the following equation holds.

$$S_{11,\text{bulk}}=\overset{12}{\underset{i=1}{\sum }}\frac{S_{11,i}}{12}$$

$$S_{21,\text{bulk}}=\overset{12}{\underset{i=1}{\sum }}\frac{S_{21,i}}{12}$$

Here, *S*_11,*i*_ and *S*_21,*i*_ are the *S* parameters for reflection and transmission of SRR_*i*_ (*i* = 1, 2, …, 12), respectively, which can be obtained by simulation. Then, the transmittance (*T*) can be calculated as *T* = |*S*_21,bulk_|^2^, and the effective refractive index (*n*_eff_) can be retrieved from the *S* parameters based on the method reported in [62].

With *a* = 200 µm, *r* = 23 µm, *w* = 15 µm, *g* = 10 µm, and *t* = 0.1 µm, simulations were performed to obtain the transmittance and effective refractive index of the calculation model containing one of the 12 types of SRRs shown in Figure 2(b). For each SRR, *y*-axis polarized wave (*θ* = 0°) or *x*-axis polarized wave (*θ* = 90°) was incident. Figure 2(c) and (d) show the transmittance and effective refractive index for a calculation model containing SRR_1_, respectively. At *θ* = 0°, the transmittance and effective refractive index were almost flat in the frequency range from 0.35 THz to 0.85 THz. This result means that SRR_1_ does not respond to the *y*-axis polarized wave in this frequency range. At *θ* = 90°, a dip in transmittance and a fluctuation in refractive index was observed at 0.686 THz. This response is a resonant response in which an electric current orbiting in the SRR is generated by the electric field of the incident wave and is called an electric response because it is caused by the electric field. Figure 2(e) and (f) show the transmittance and effective refractive index for a calculation model containing SRR_2_. As in the case of the electric response, a dip in transmittance and a fluctuation in refractive index was observed. On the other hand, the resonant frequency was 0.683 THz, and the SRR responded to *y*-axis polarized wave (*θ* = 0°) while it did not respond to *x*-axis polarized wave (*θ* = 90°). This response is also a resonant response that generates an orbital current in the SRR. However, unlike the electric response, the orbital current is generated by the magnetic field penetrating the SRR; therefore, it is called a magnetic response. The above results confirmed that the SRRs with the designed dimensions show both electric and magnetic responses around 0.683–0.686 THz.

In the calculation model shown in Figure 2(b), the 12 types of SRRs respond differently to the electromagnetic field. For example, SRR_2_, SRR_4_, SRR_6_, SRR_8_, SRR_10_, and SRR_12_ respond to *y*-axis polarized wave (*θ* = 0°) although they respond by different mechanisms; SRR_2_ and SRR_4_ show magnetic responses, SRR_6_ and SRR_8_ show electric responses, and SRR_10_ and SRR_12_ show their combined responses. On the other hand, for *x*-axis polarized wave (*θ* = 90°), SRR_1_ and SRR_3_ show electric responses, SRR_5_ and SRR_7_ show magnetic responses, and SRR_9_ and SRR_11_ show their complex responses. Therefore, if the 12 types of SRRs are evenly included in the bulk metamaterial, 6 types of SRRs resonate regardless of the polarization, and a polarization-independent response is expected. Figure 2(g) and (h) show the transmittance and effective refractive index for the bulk metamaterial, respectively, which was obtained from the average *S* parameters of 12 types of SRRs. The results with *x*-axis and *y*-axis polarized waves were almost the same, which means that the polarization dependence was eliminated. A dip in transmittance and a fluctuation in refractive index were observed at 0.685 THz, which was between the resonant frequency of magnetic response, 0.683 THz, and that of electric response, 0.686 THz, although the dip and fluctuation were smaller than those shown in Figures 2(c)–(f). This is probably because not all types of SRRs respond to incident electromagnetic fields in the bulk metamaterial, and the average resonance response is weaker than when only one type of SRRs is included.

### 2.2 Fabrication

**Figure 3:**
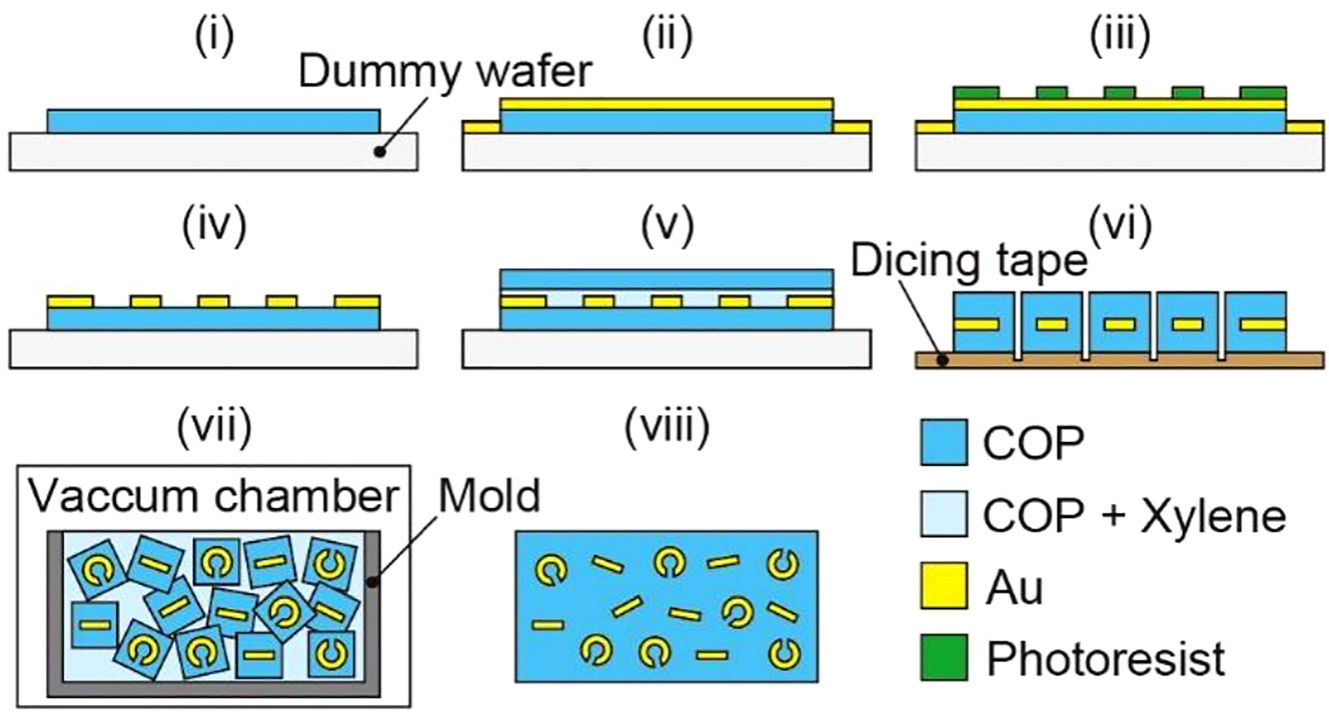
Fabrication processes for the proposed 3D bulk metamaterial.

Resin grains containing the designed SRRs and a 3D bulk metamaterial were fabricated. Figure 3 shows the fabrication processes. First, a square COP film (ZeonorFilm ZF14, Zeon Corporation) with a side length of 80 mm and a thickness of 100 μm was fixed on a 5-inch dummy silicon wafer using Kapton polyimide tape. Second, gold with a thickness of 100 nm was deposited on the COP film by sputtering. Third, photoresist (OFPR-800 34cp, Tokyo Ohka Kogyo Co., Ltd.) was spin-coated on the gold layer at 2000 rpm for 20 s, and SRR patterns of the photoresist were formed by photolithography in a square area with a side length of 60 mm. Fourth, the gold layer was etched with a gold etchant to obtain gold SRR patterns. Fifth, a solution in which 20 wt% of COP pellets (Zeonex 480R, Zeon Corporation) was dissolved in xylene was spin-coated on the COP film with gold SRRs at 2000 rpm for 20 s, and a COP film with a side length of 60 mm and a thickness of 100 μm was layered. Then, the sample was put into a vacuum pack, which was evacuated and left for 24 h to bond the upper COP film to the lower COP film with gold SRRs sandwiched between them; thus, a resin sheet containing SRRs was fabricated. Sixth, the resin sheet was transferred from the dummy wafer to a dicing tape (Adwill G-19, LINTEC Corporation) and diced under conditions such that the desired grain size was obtained. For example, if the distance between the centers of adjacent SRRs formed within the resin sheet was set to 225 μm and diced with a blade width of 20 μm, the kerf width became approximately 25 μm, so that grains with a size of approximately 200 μm could be obtained. Dicing was performed using an automatic dicing saw (DAD3350, DISCO Corporation) with an electroformed bond hubless blade (Z09-SD1700-Y1-120 50.4x0.02A1x40, DISCO Corporation). The rotation speed of the spindle, the feed rate of the blade, and the amount of cut into the tape were set to 30,000 rpm, 10 mm/s, and 50 μm, respectively. After dicing, the tape was soaked in acetone to remove the adhesive between the tape and the resin grains, and the grains were separated from the tape. Seventh, the grains were filled in a cylindrical mold with a thickness of 4 mm and a diameter of 12 mm, and a 15 wt% COP solution dissolved in xylene was poured into the mold. Then, the mold was placed in a vacuum chamber to defoam and solidify the COP solution. After complete solidification, the bulk metamaterial was obtained by removing it from the mold. Since the top and bottom surfaces of the bulk metamaterial after removal from the mold were uneven, polishing was performed so that it possessed the desired thickness and flat surfaces.

**Figure 4:**
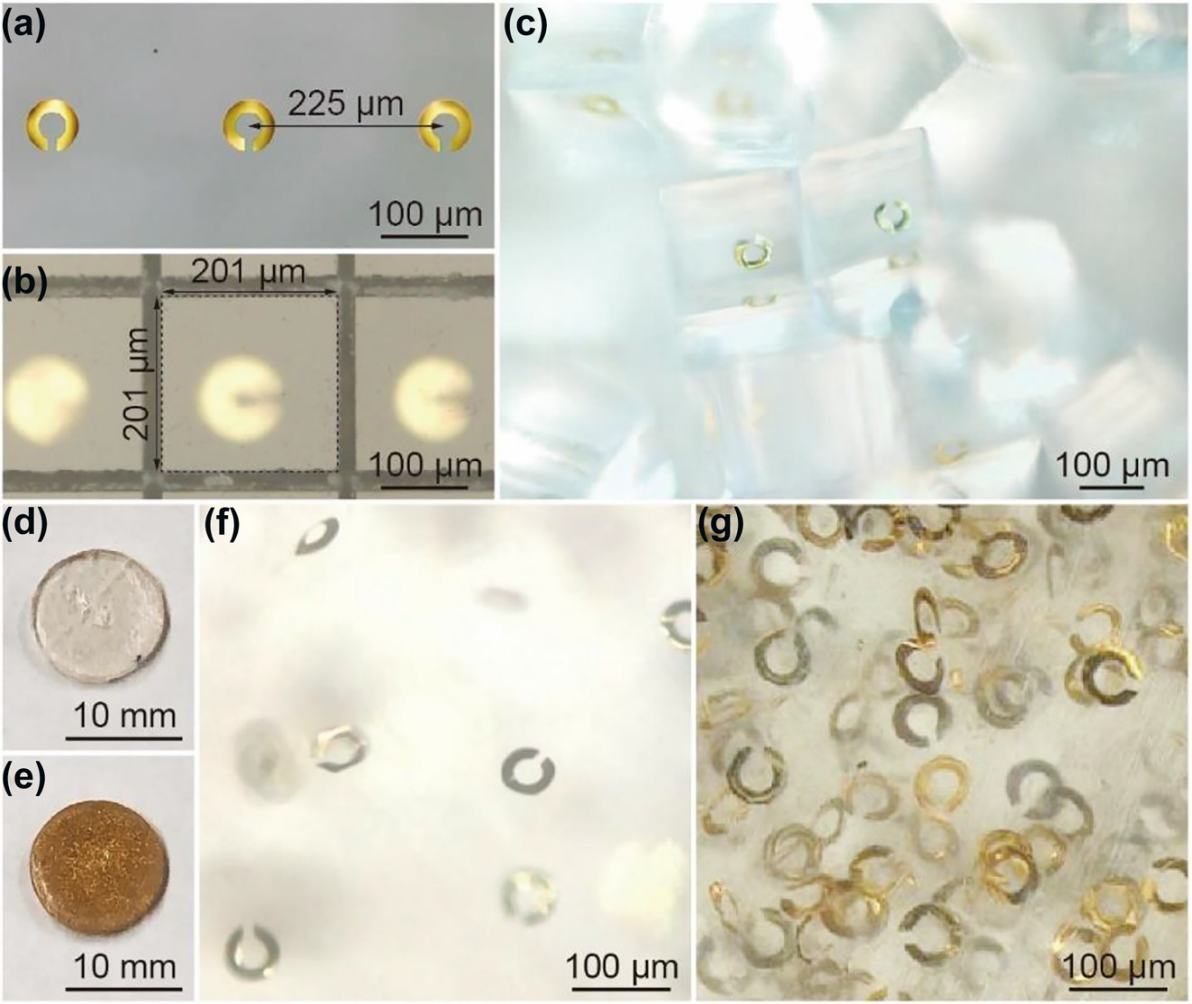
(a)–(c) Enlarged views of the resin sheet containing SRRs, the resin sheet on the dicing tape after the dicing process, and the grains after removing from the dicing tape, respectively. (d) and (e) Overviews of the bulk metamaterials fabricated using 200 µm and 100 µm grains, respectively. (f) and (g) Enlarged views of the bulk metamaterials shown in (d) and (e), respectively.

Figure 4(a) shows an enlarged view of the fabricated resin sheet containing SRRs. The distance of the centers of adjacent SRRs was 225 µm. The radius (*r*), line width (*w*), and gap width (*g*) of the fabricated SRR were 22 µm, 14 µm, and 10 µm, respectively. The fabrication error was 1 μm or less, and the SRRs were fabricated almost as designed. Figure 4(b) shows an enlarged view of the resin sheet on the dicing tape after dicing. The focus was on the upper surface of the resin sheet. The kerfs were confirmed to have a square shape, and the grain size was 201 μm on each side of the square. The design value of the grain size was 200 μm. Therefore, the fabrication error was 1 μm, that is, 0.5% of the grain size. Since the fabrication error related to the grain size depends on the accuracy of the dicing process, it is important to set the conditions for proper processing of COP films. Figure 4(c) shows the resin grains containing SRRs after removal from the dicing tape. An SRR was contained in a cubic resin grain. In a resin sheet in which SRRs were formed at intervals of 225 μm in a 60 mm square region, 70,756 cubic grains with a side length of 200 μm could be obtained. Collecting them all gives a volume of approximately 566 mm^3^. The mold used in this study was a cylinder with a diameter of 12 mm and a thickness of 4 mm, and the volume was approximately 452 mm^3^. Therefore, it was possible to fill the mold with grains made using one dummy wafer. To efficiently produce grains, it will be necessary to maximize the number of grains to be fabricated on one dummy wafer in the future. Figure 4(d) shows the fabricated bulk metamaterial after it was removed from the mold and polished. The bulk metamaterial possessed a diameter of 12 mm, and a thickness of 1.6 mm and was almost transparent. Figure 4(e) shows a bulk metamaterial made using grains with the same dimensions of SRRs and a grain size of 100 μm. In fabricating these grains, COP films with a thickness of 50 μm were used. The diameter and thickness were the same as those of the bulk metamaterial using 200 μm grains. Bulk metamaterials with 100 μm grains should contain SRRs that are eight times denser than bulk metamaterials with 200 μm grains. The transparency of the bulk metamaterial fabricated using 100 μm grains was lower than that of the bulk metamaterial fabricated using 200 μm grains, and it contained more gold SRRs. Figure 4(f) and (g) show enlarged views of the bulk metamaterial fabricated using 200 μm grains shown in Figure 4(d) and 100 μm grains shown in Figure 4(e), respectively. It was confirmed that both of the bulk metamaterials contained SRRs dispersed in random directions, and that the density of the bulk metamaterial with 100 μm grains was higher than that of the bulk metamaterial with 200 μm grains.

## 3 Optical properties

To evaluate the optical properties of the fabricated bulk metamaterials, transmittance measurements were performed using a terahertz time-domain spectroscopy (THz-TDS) system (THZ-TDS2000ms, NIPPO PRECISION Co. Ltd.). In THz-TDS, measurement samples are irradiated with a terahertz wave pulse, and the waveforms are measured when the wave pulse passes through the sample and when there is no sample as a reference. Then, each waveform is Fourier transformed, and the spectra of transmittance and phase delay are obtained. The frequency resolution was approximately 9.16 GHz. The effective refractive index of the bulk metamaterial is calculated from the phase delay and the thickness of bulk metamaterial. All samples were set to be perpendicular to the traveling direction of the terahertz wave. To evaluate the polarization dependence, after the first measurement of each sample, the polarization direction with respect to the sample was changed by 90°, and the measurement was performed again.

**Figure 5:**
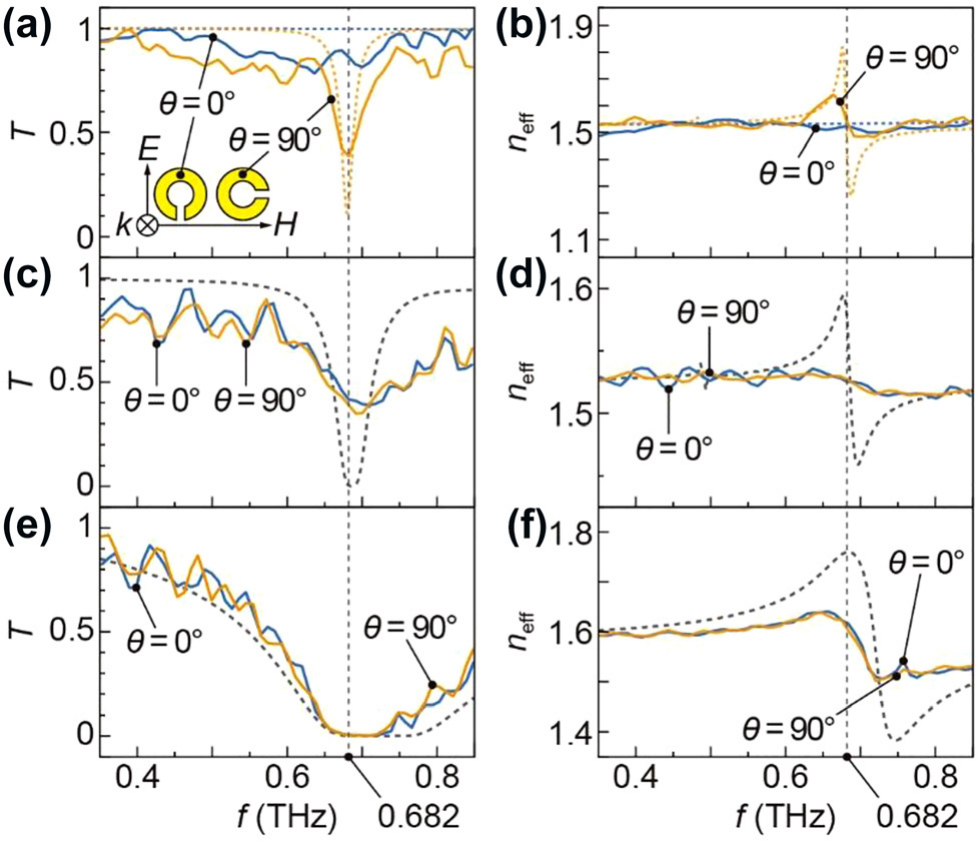
(a) and (b) The transmittance spectra and the effective refractive index of the resin sheet containing SRRs for 200 μm grains, respectively. The inset shows the definition of the incident polarization angle to the direction of SRRs. (c) and (d) The transmittance spectra and the effective refractive index of the bulk metamaterial using 200 μm grains. (e) and (f) The transmittance spectra and the effective refractive index of the bulk metamaterial using 100 μm grains. For all figures, dashed lines show the simulation results.

First, the transmittance and effective refractive index of the resin sheet containing SRRs before dicing were measured. Figure 5(a) shows the measured and simulated transmittance spectra of the resin sheet used to fabricate 200 μm grains. The incident polarization angle was defined with respect to the direction of the SRR formed in the resin sheet, as shown in the inset of Figure 5(a). Note that the simulation result is different from one shown in Figure 2(c) and (d) because the kerf width exists in the state of the resin sheet, and the size of the unit cell (*a*) was considered as 225 μm. At *θ* = 0°, the transmittance spectrum was almost flat, and there was no resonant response as in the simulation result. The slight decrease in transmittance at approximately 0.5 THz seemed to be due to the effect of interference inside the sheet. At *θ* = 90°, a dip was confirmed at 0.682 THz, which was in good agreement with the resonant frequency of the electric response of SRRs confirmed in the simulation, 0.680 THz. The sharpness of the dip in the measurement result is worse than that in the simulation result; the minimum transmittance was from 11.2% to 38.7%, and the full width at half maximum was from 0.019 THz to 0.064 THz. Figure 5(b) shows the measured and simulated effective refractive index of the resin sheet. The spectrum was flat at *θ* = 0°, while the fluctuation of the refractive index was confirmed at the resonant frequency at *θ* = 90°. In the simulation result, the maximum value of the refractive index was 1.808 at 0.676 THz, and the minimum value was 1.273 at 0.688 THz. On the other hand, in the measurement result, the maximum value of the refractive index was 1.640 at 0.664 THz, and the minimum value was 1.482 at 0.719 THz; thus, the fluctuation range was 0.158, which was smaller than that in the simulation result, 0.535. A possible cause of the deterioration in the sharpness of transmittance and the fluctuation range of refractive index is the dielectric loss of COP, which was not considered in the simulation. Another possible cause is the fabrication error in the thickness of the gold layer due to nonuniformity in sputtering deposition.

Next, the transmittance and effective index of the bulk metamaterials were measured. Figure 5(c) shows the measured and simulated transmittance spectra of the bulk metamaterial using 200 μm grains. Since the thickness of the simulation model was 200 μm, in order to compare with the measurement result with a thickness of 1.6 mm, the transmittance of the simulation result was raised to the 8th power. At *θ* = 0°, a dip was confirmed at approximately 0.682 THz, which was broader and shallower than that in the simulation result. The frequency at which the transmittance was minimized was slightly lower in the simulation result than in the measurement result. This is probably because the arrangement of SRRs in the simulation model was different from the actual bulk metamaterial in which SRRs were randomly dispersed in a bulk COP with different grain-to-grain spacing. The spectrum at *θ* = 90° was almost the same as that at *θ* = 0°, which indicates that the polarization dependence seen in the sheet state has disappeared. Figure 5(d) shows the measured and simulated effective refractive index of the bulk metamaterial using 200 μm grains. There was fluctuation at approximately 0.682 THz at both *θ* = 0° and 90°, which was also confirmed in the simulation. In the frequency range from 0.63 THz to 0.75 THz, the refractive indices ranged from 1.514 at 0.737 THz to 1.536 at 0.636 THz in the measurement result at *θ* = 0° and from 1.459 at 0.697 THz to 1.594 at 0.678 THz in the simulation result; therefore, the fluctuation ranges were 0.022 in the measurement result and 0.135 in the simulation result. The fluctuation range was smaller in the measurement result than in the simulation result in both the sheet state and the bulk metamaterial. While the fluctuation ranges around the resonant frequency differed, the refractive indices at 0.35 THz and 0.85 THz were in good agreement between the measurement and simulation results. This result indicates that the simulation can predict the properties of bulk metamaterials even when using a simple calculation model that does not included countless randomly distributed SRRs, as in real bulk metamaterials.

Figure 5(e) shows the measured and simulated transmittance spectra of the bulk metamaterial using 100 μm grains. Since the thickness of the simulation model was 100 μm, in order to compare with the measurement result with a thickness of 1.6 mm, the transmittance of the simulation result was raised to the 16th power. As with the bulk metamaterial using 200 μm grains, both measurement and simulation results showed a decrease in transmittance near the resonant frequency of 0.682 THz, and the spectrum was polarization independent. On the other hand, compared to the bulk metamaterial using 200 μm grains, the transmittance at the resonant frequency is smaller in the bulk metamaterial using 100 μm grains, even though the sample thickness was the same. The minimum transmittance was 0.13% at 0.711 THz at *θ* = 0°. This result indicates that increasing the density of SRRs in bulk metamaterials reduces the transmittance. Figure 5(f) shows the measured and simulated effective refractive index of the bulk metamaterial using 100 μm grains. As with the bulk metamaterial using 200 μm grains, both measurement and simulation results showed a fluctuation in refractive index near the resonant frequency of 0.682 THz, even though its range was smaller in the measurement result than in the simulation result. In the frequency range from 0.63 THz to 0.75 THz, the refractive indices ranged from 1.506 at 0.730 THz to 1.641 at 0.657 THz in the measurement result at *θ* = 0° and from 1.383 at 0.746 THz to 1.760 at 0.684 THz in the simulation result; therefore, the fluctuation ranges were 0.135 in the measurement result and 0.377 in the simulation result. The fluctuation range of the bulk metamaterial using 100μm grains was 6.1 times that of the bulk metamaterial using 200 μm grains in the measurement result and 2.8 times in the simulation result. In addition, at 0.35 THz, the refractive index of the bulk metamaterial using 100-μm grains was approximately 1.592 while that of the bulk metamaterial using 200 μm grains was 1.529; thus, the refractive index in the low-frequency region increased by 0.063. These results indicate that increasing the density of SRRs in bulk metamaterials increases the refractive index in the low-frequency region as well as the fluctuation range around the resonant frequency. At 0.85 THz, the refractive index of the bulk metamaterial using 100 μm grains was approximately 1.525 while that of the bulk metamaterial using 200 μm grains was 1.514.

In this study, we designed and fabricated a 3D bulk metamaterial with the aim of realizing a terahertz optical material with a refractive index that cannot be obtained in nature. From the measurement results, it was found that by randomly dispersing SRRs in bulk COP, the refractive index in the low-frequency region can be increased, and fluctuations can occur near the resonant frequency of the SRRs. The maximum fluctuation range was 0.135, and this fluctuation occurred within a narrow frequency range of approximately 0.06 THz from 0.684 THz to 0.746 THz. This characteristic indicates the possibility of achieving a spectroscopic prism with a large dispersion in this frequency range. Furthermore, the fabricated bulk metamaterial showed independence from polarization, which indicates that the anisotropy shown in the resin sheet was eliminated in the bulk metamaterial. These results show that by dispersing metamaterials in bulk COP, it is possible to control the original refractive index of COP to something different. On the other hand, considering the decreased in transmittance, it needs to be improved before the proposed bulk metamaterial can be used as an optical material. Much of the nontransmitted energy is reflected, probably due to the impedance mismatch between the bulk metamaterial and the incident medium. One possible solution is to change the shape of the metamaterial to a different shape which shows both high transmittance and a high refractive index. In the field of metasurfaces, structures that have both high transmittance and high refractive index have been reported [47], and it is conceivable to apply these structures to 3D bulk metamaterials.

## 4 Conclusions

We proposed isotropic 3D bulk metamaterials made by a novel method that solidifies grains obtained by dicing a resin sheet containing metal microstructures. The simulation and fabrication when SRRs were selected as the microstructures were demonstrated, and the optical properties of the fabricated bulk metamaterial were measured. From the measurement results, it was found that the fabricated bulk metamaterial showed the maximum fluctuation range of the refractive index of 0.135 near the resonant frequency of the included SRRs, and the refractive index increased in the low-frequency region. The fabrication method shown in this study can be applied to the shapes of metamaterials other than SRRs and may provide a wide range of choices for terahertz optical materials in the future.

## References

[1] Mittleman D. M. (2013). Frontiers in terahertz sources and plasmonics. *Nat. Photonics*.

[2] Ferguson B., Zhang X. (2002). Materials for terahertz science and technology. *Nat. Mater.*.

[3] Beard M. C., Turner G. M., Schmuttenmaer C. A. (2002). Terahertz spectroscopy. *J. Phys. Chem. B*.

[4] Siegel P. H. (2002). Terahertz technology. *IEEE Trans. Microw. Theor. Tech.*.

[5] F Federici J., Schulkin B., Huang F. (2005). THz imaging and sensing for security applications—explosives, weapons and drugs. *Semicond. Sci. Technol.*.

[6] Tonouchi M. (2007). Cutting-edge terahertz technology. *Nat. Photonics*.

[7] Davies A. G., Burnett A. D., Fan W., Linfield E. H., Cunningham J. E. (2008). Terahertz spectroscopy of explosives and drugs. *Mater. Today*.

[8] Jepsen P. U., Cooke D. G., Koch M. (2011). Terahertz spectroscopy and imaging – modern techniques and applications. *Laser Photon. Rev.*.

[9] Ulbricht R., Hendry E., Shan J., Heinz T. F., Bonn M. (2011). Carrier dynamics in semiconductors studied with time-resolved terahertz spectroscopy. *Rev. Mod. Phys.*.

[10] Kampfrath T., Tanaka K., Nelson K. A. (2013). Resonant and nonresonant control over matter and light by intense terahertz transients. *Nat. Photonics*.

[11] Dhillon S. S., Vitiello M. S., Linfield E. H. (2017). The 2017 terahertz science and technology roadmap. *J. Phys. Appl. Phys.*.

[12] Kawase K., Ogawa Y., Watanabe Y., Inoue H. (2003). Non-destructive terahertz imaging of illicit drugs using spectral fingerprints. *Opt. Express*.

[13] Optical glass Edmund optics. ..

[14] Rogalin V. E., Kaplunov I. A., Kropotov G. I. (2018). Optical materials for the THz range. *Opt. Spectrosc.*.

[15] Naftaly M., Miles R. E. (2007). Terahertz time-domain spectroscopy of silicate glasses and the relationship to material properties. *J. Appl. Phys.*.

[16] Dai J., Zhang J., Zhang W., Grischkowsky D. (2004). Terahertz time-domain spectroscopy characterization of the far-infrared absorption and index of refraction of high-resistivity, float-zone silicon. *J. Opt. Soc. Am. B*.

[17] Podzorov A., Gallot G. (2008). Low-loss polymers for terahertz applications. *Appl. Opt.*.

[18] Kanamori Y. (2021). High-efficiency optical filters based on nanophotonics. *IEEJ Trans. Electr. Electron. Eng.*.

[19] Kabashin A. V., Evans P., Pastkovsky S. (2009). Plasmonic nanorod metamaterials for biosensing. *Nat. Mater.*.

[20] Liu N., Weiss T., Mesch M. (2010). Planar metamaterial analogue of electromagnetically induced transparency for plasmonic sensing. *Nano Lett.*.

[21] Liu N., Mesch M., Weiss T., Hentschel M., Giessen H. (2010). Infrared perfect absorber and its application as plasmonic sensor. *Nano Lett.*.

[22] Ebrahimi A., Withayachumnankul W., Al-Sarawi S., Abbott D. (2014). High-sensitivity metamaterial-inspired sensor for micro-fluidic dielectric characterization. *IEEE Sensor. J.*.

[23] Singh R., Cao W., Al-Naib I., Cong L., Withayachumnankul W., Zhang W. (2014). Ultrasensitive terahertz sensing with high-*Q* Fano resonances in metasurfaces. *Appl. Phys. Lett.*.

[24] Kanamori Y., Shimizu T., Nomura S.-I., Hane K. (2019). Fabrication of refractive index sensors using polarization independent metamaterial and their application to chemical concentration measurement and DNA detection. *Electron. Commun. Jpn.*.

[25] Okatani T., Sekiguchi S., Hane K., Kanamori Y. (2020). Surface-plasmon-coupled optical force sensors based on metal-insulator-metal metamaterials with movable air gap. *Sci. Rep.*.

[26] Chen H.-T., Padilla W. J., Zide J. M. O., Gossard A. C., Taylor A. J., Averitt R. D. (2006). Active terahertz metamaterial devices. *Nature*.

[27] Chen H.-T., Padilla W. J., Cich M. J., Azad A. K., Averitt R. D., Taylor A. J. (2009). A metamaterial solid-state terahertz phase modulator. *Nat. Photonics*.

[28] Gu J., Singh R., Liu X. (2012). Active control of electromagnetically induced transparency analogue in terahertz metamaterials. *Nat. Commun.*.

[29] Sun Z., Martinez A., Wang F. (2016). Optical modulators with 2D layered materials. *Nat. Photonics*.

[30] Zhang Y., Liu H., Cheng H., Tian J., Chen S. (2020). Multidimensional manipulation of wave fields based on artificial microstructures. *Opto-Electron. Adv.*.

[31] Huang Y., He Q., Zhang D., Kanamori Y. (2021). Switchable band-pass filter for terahertz waves using VO_2_-based metamaterial integrated with silicon substrate. *Opt. Rev.*.

[32] Shrekenhamer D., Chen W.-C., Padilla W. J. (2013). Liquid crystal tunable metamaterial absorber. *Phys. Rev. Lett.*.

[33] Ishii Y., Takida Y., Kanamori Y., Minamide H., Hane K. (2017). Fabrication of metamaterial absorbers in THz region and evaluation of the absorption characteristics. *Electron. Commun. Jpn.*.

[34] Kanamori Y. (2015). Wavelength selective filters using metamaterials in terahertz frequency region. *Proceedings of 2015 IEEE International Symposium on Radio-Frequency Integration Technology*.

[35] Ema D., Kanamori Y., Sai H., Hane K. (2018). Plasmonic color filters integrated on a photodiode array. *Electron. Commun. Jpn.*.

[36] Wang D., Zhang L., Gu Y. (2015). Switchable ultrathin quarter-wave plate in terahertz using active phase-change metasurface. *Sci. Rep.*.

[37] Okatani T., Abe Y., Nakazawa T., Hane K., Kanamori Y. (2021). Fabrication of silicon nanospheres placeable on a desired position for dielectric metamaterials in the visible region. *Opt. Mater. Express*.

[38] Kanamori Y., Hokari R., Hane K. (2015). MEMS for plasmon control of optical metamaterials. *IEEE J. Sel. Top. Quant. Electron.*.

[39] Zhao X., Duan G., Li A., Chen C., Zhang X. (2019). Integrating microsystems with metamaterials towards metadevices. *Microsyst. Nanoeng.*.

[40] Chang Y., Wei J., Lee C. (2020). Metamaterials – from fundamentals and MEMS tuning mechanisms to applications. *Nanophotonics*.

[41] Huang Y., Nakamura K., Takida Y., Minamide H., Hane K., Kanamori Y. (2020). Actively tunable THz filter based on an electromagnetically induced transparency analog hybridized with a MEMS metamaterial. *Sci. Rep.*.

[42] Liu J., Shi M., Chen Z., Wang S., Wang Z., Zhu S. (2021). Quantum photonics based on metasurfaces. *Opto-Electron. Adv.*.

[43] Pendry J. B., Holden A. J., Robbins D. J., Stewart W. J. (1999). Magnetism from conductors and enhanced nonlinear phenomena. *IEEE Trans. Microw. Theor. Tech.*.

[44] Shelby R. A., Smith D. R., Schultz S. (2001). Experimental verification of a negative index of refraction. *Science*.

[45] Suzuki T., Sekiya M., Sato T., Takebayashi Y. (2018). Negative refractive index metamaterial with high transmission, low reflection, and low loss in the terahertz waveband. *Opt. Express*.

[46] Suzuki T., Asada H. (2020). Reflectionless zero refractive index metasurface in the terahertz waveband. *Opt. Express*.

[47] Asada H., Endo K., Suzuki T. (2021). Reflectionless metasurface with high refractive index in the terahertz waveband. *Opt. Express*.

[48] Huang Y., Kosugi A., Naito Y. (2021). Improvement in THz light extraction efficiencies with antireflection subwavelength gratings on a silicon prism. *Jpn. J. Appl. Phys.*.

[49] Zhang X., Liu Z. (2008). Superlenses to overcome the diffraction limit. *Nat. Mater.*.

[50] Tyc T., Zhang X. (2011). Perfect lenses in focus. *Nature*.

[51] Glybovski S. B., Tretyakov S. A., Belov P. A., Kivshar Y. S., Simovski C. R. (2016). Metasurfaces: from microwaves to visible. *Phys. Rep.*.

[52] Lalanne P., Chavel P. (2017). Metalenses at visible wavelengths: past, present, perspectives. *Laser Photon. Rev.*.

[53] Soukoulis C. M., Wegener M. (2011). Past achievements and future challenges in the development of three-dimensional photonic metamaterials. *Nat. Photonics*.

[54] Paul O., Imhof C., Reinhard B., Zengerle R., Beigang R. (2008). Negative index bulk metamaterial at terahertz frequencies. *Opt. Express*.

[55] Han N. R., Chen Z. C., Lim C. S., Ng B., Hong M. H. (2011). Broadband multi-layer terahertz metamaterials fabrication and characterization on flexible substrates. *Opt. Express*.

[56] Vignolini S., Yufa N. A., Cunha P. S. (2012). A 3D optical metamaterial made by self-assembly. *Adv. Mater.*.

[57] Liu N., Guo H., Fu L., Kaiser S., Schweizer H., Giessen H. (2008). Three-dimensional photonic metamaterials at optical frequencies. *Nat. Mater.*.

[58] Gansel J. K., Thiel M., Rill M. S. (2009). Gold helix photonic metamaterial as broadband circular polarizer. *Science*.

[59] Burckel D. B., Wendt J. R., Ten Eyck G. A. (2010). Micrometer-scale cubic unit cell 3D metamaterial layers. *Adv. Mater.*.

[60] Chen C.-C., Ishikawa A., Tang Y.-H., Shiao M.-H., Tsai D. P., Tanaka T. (2015). Uniaxial-isotropic metamaterials by three-dimensional split-ring resonators. *Adv. Opt. Mater.*.

[61] Fan K., Strikwerda A. C., Zhang X., Averitt R. D. (2013). Three-dimensional broadband tunable terahertz metamaterials. *Phys. Rev. B*.

[62] Chen X., Grzegorczyk T. M., Wu B.-I., Pacheco J., Kong J. A. (2004). Robust method to retrieve the constitutive effective parameters of metamaterials. *Phys. Rev.*.

